# Learning Participation and Performance Among Social and Academic Learners in a Nursing Massive Open Online Course: Retrospective Cohort Study

**DOI:** 10.2196/90827

**Published:** 2026-06-02

**Authors:** Jianqing Zeng, Kun Li, Wenxuan Wang, Lingjun Xiao, Yingmin Wang, Xinghui He, Yonghong Qing, Jingjing Xue, Lirong Zhong, Tiebin Yan, Xiuyuan Zheng

**Affiliations:** 1 School of Nursing, Sun Yat-sen University Guangzhou China; 2 Sun Yat-sen Memorial Hospital, Sun Yat-sen University Guangzhou China

**Keywords:** massive open online courses, MOOCs, nursing education, learning participation, academic performance, latent class analysis

## Abstract

**Background:**

Massive open online courses (MOOCs) are increasingly used in nursing education, attracting learners with diverse participation modes. Social learners (self-directed) enroll voluntarily based on personal interests, while academic learners (institution-directed) are mandated by nursing schools, with performance linked to grades. Existing literature shows that academic learners outperform social learners in MOOCs, but evidence is scarce on whether this performance gap stems from distinct participation patterns shaped by institutional requirements.

**Objective:**

We sought to compare participation typologies between social and academic learners using latent class analysis (LCA) and assess their association with learning outcomes.

**Methods:**

A retrospective analysis was conducted of 8222 students enrolled in a rehabilitation nursing MOOC from 2021 to 2022. Learners were classified by topic test participation frequency via LCA, and their distributions and scores were statistically compared.

**Results:**

The cohort comprised 7683 (93.4%) academic and 539 (6.6%) social learners. LCA identified 3 participation types: participation-declining (93/8222, 1.1%), high-participation (6959/8222, 84.6%), and lowest-participation (1170/8222, 14.2%). The distributions of participation types differed significantly between groups (*χ*^2^=2745.1; *df*=2; *P*<.001). Most social learners belonged to the lowest-participation group (482/539, 89.4%), whereas most academic learners belonged to the high-participation group (6923/7683, 90.1%). Among high-participation learners, academic learners significantly outperformed social learners (all *P*<.05; *r=*0.06-0.10). No significant between-group differences were observed for the other participation types (all *P*>.05).

**Conclusions:**

Distinct participation patterns were associated with the performance advantage of academic learners. Our findings highlight the importance of sustained participation, suggesting that MOOC instructors should provide enhanced support for voluntary learners.

## Introduction

Massive open online courses (MOOCs) have gained widespread attention in nursing education due to their openness and accessibility [1]. MOOCs provide unprecedented opportunities for lifelong learning and professional development and, over recent years, have been widely introduced into nursing education, becoming a key approach for promoting reform in nursing teaching and enhancing the professional competence of nursing staff [2,3]. However, while providing convenience, MOOCs also face a common challenge relating to a high enrollment rate but a low completion rate, with significant variations in the participation of learners and their learning performance [4]. Studies have shown that the initial motivation of learners is a key factor influencing their online learning behaviors and final outcomes [5]. On the basis of motivation, learners are categorized as “social learners” (voluntary) and “academic learners” (institution-mandated), with their performance often linked to credits or final grades and driven by significant external motivations [6].

Previous studies indicate that academic learners often exhibit higher participation than social learners due to external motivation [7], which confirms the important role of external motivation in maintaining online learning behaviors. However, these studies have overlooked the heterogeneity and complexity within groups of different learners. For example, social or mixed MOOC learners were classified as committed, early dropout, and other learners [8]; Wang et al [9] used latent class analysis (LCA) to categorize MOOC learners into committed, negative, midterm dropout, and early dropout learner types. Not all academic learners are actively engaged, nor are all social learners passively inactive: both groups include learners with high participation, minimal effort, and midterm withdrawal. This heterogeneity within each group suggests that a simple division of social and academic learners cannot fully explain the performance differences in MOOCs [8].

Therefore, simply applying “social” or “academic” distinctions to explain differences in learning performance may obscure the real factors associated with this phenomenon. We need to consider whether the improved overall performance of academic learners due to the “academic” identity can improve their efficiency of learning or whether the proportion of “high-participation learners” within the academic learner group is much higher than that in the social learner group. If learners can be objectively classified based on their actual online learning behavioral characteristics, and then the distribution differences of learners with different backgrounds in these behavioral categories can be compared, it may be possible to more accurately identify the key factors associated with learning performance.

Consequently, in this study, we retrospectively analyzed data from a rehabilitation nursing MOOC and applied LCA to identify different participation types based on the participation behaviors of learners in topic tests. We analyzed and compared differences in the proportion of these participation types between social and academic learners and investigated how these differences in distribution might be associated with final learning performance. In this study, we aimed to comprehensively analyze the heterogeneity within learner groups to provide empirical evidence for nursing educators to design more targeted MOOC teaching strategies and implement refined teaching management, thereby effectively improving the quality and efficacy of online nursing education.

## Methods

We conducted a retrospective study using data from a rehabilitation nursing MOOC.

### Participants

This MOOC featured a dual structure. First, the course was freely open to the public without prerequisites. Second, participants who registered and engaged voluntarily were defined as social learners. Learners assigned by university teachers, who received blended online and offline instruction, were defined as academic learners with relatively smaller class sizes.

**Figure 1:**
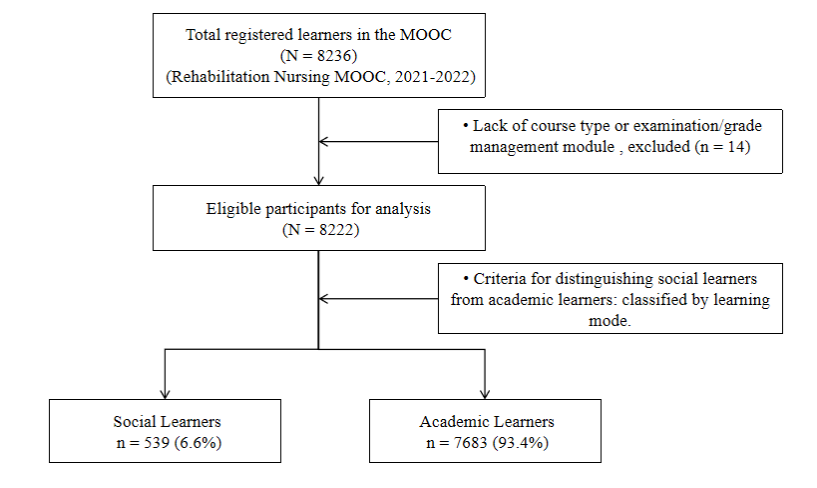
Flowchart of study participant screening. MOOC: massive open online courses.

Our dataset included all social and academic learners registered across 3 semesters (2021-2022) who had complete data on course type and examination and grade management modules. After excluding learners with missing information, the final sample comprised 8222 participants (Figure 1). This sample size far exceeds the minimum requirement of 300 for valid category analysis [10], ensuring sufficient statistical power. Notably, the online learning platform did not collect demographic characteristics (such as age, gender, and prior educational background) during learner registration, precluding control for these potential confounding variables.

### The Rehabilitation Nursing MOOC

The rehabilitation nursing MOOC was hosted on the Wisdom Tree online education platform [11]. The primary objective of this course was to enhance the comprehension of rehabilitation nursing theory through perceptual learning. The course welcomed all who registered via the Wisdom Tree platform without imposing any prerequisites. The course featured patient evaluation, rehabilitation nursing evaluation, rehabilitation treatment technology, rehabilitation nursing technology, and rehabilitation nursing for common diseases. Academic learners were provided with offline direct feedback, offline practicum activities, and online assessments. Offline direct feedback included face-to-face consultation between teachers and students and detailed feedback on assignments, provided every 2 weeks. Offline practical activities, arranged every 4 weeks in line with the teaching schedule, involved practical training related to the specialty. A diverse range of online assessments was used to evaluate the knowledge mastery of each learner. Diverse instructional resources, such as teaching videos, topic-specific assessments, and downloadable teaching materials, were used to deliver the content of each topic. Topic assessments comprised 5 to 17 questions that aimed to evaluate the understanding of each learner in that specific area.

Limitations in platform data collection restricted our analysis to topic test participation as the sole measure of learning participation; broader engagement metrics (eg, logins, video viewing duration, time-on-task, forum activity, and teaching material downloads) were not available for extraction and analysis.

### Outcome Indicators

Learners who completed and submitted topic tests (including partial attempts and 0 scores) were treated as participants for each topic. The participation rate in each topic test was computed by dividing the number who submitted a test (achieved a score, including 0) by the total number enrolled. The learner types were then categorized via LCA based on data from 6 administered topic tests.

Learning performance was the secondary outcome indicator, quantified by individual topic test scores. Because the 6 tests had different numbers of questions, raw scores were standardized to a 0 to 100 scale by dividing the actual score by the maximum possible score and multiplying by 100.

### Data Analysis

All statistical analyses were performed using SPSS software (version 25.0; IBM Corp) and Mplus software (version 8.3; Muthén & Muthén). Descriptive statistics were computed to describe the number of social and academic learners considering each topic test, including participation rates, frequencies, and percentages.

LCA quantifies the heterogeneity of a given population by classifying individuals based on their different response patterns across a series of observed indicators. For LCA in this study, we used binary indicator coding (1=submitted the topic test and 0=did not submit the topic test). Model estimation was conducted via maximum likelihood with full information maximum likelihood to handle missing data, adhering to the local independence assumption of LCA. We applied LCA to categorize learners based on their participation in each topic test. The distribution of social learners and academic learners across different learning participation types was computed, and results were expressed as frequencies and percentages. Differences were then tested for significance using the chi-square test. Descriptive statistics were also computed for the scores acquired by social and academic learners in each topic test. Scores are represented as means and SDs. The significance of the disparities in performance between social learners and academic learners was evaluated using 2-tailed *t* tests (for normally distributed data) or Mann-Whitney *U* tests (for data that were not normally distributed). Because of the limited reliability and stability of statistical analyses, comparisons between subgroups with small sample sizes (n<5) and the other 2 groups were considered exploratory.

To ascertain the optimum number of latent classes, a single-class model was first prepared as a baseline for comparison. Subsequently, the number of latent classes was incrementally increased, and the fit indices of the resulting models were compared. The best model, with the optimum number of classes, was determined based on both statistical and practical significance [12]. We considered several indices of model fit, including the Akaike information criterion, Bayesian information criterion, and the adjusted Bayesian information criterion. For each of these indices, a smaller value indicates a better model fit. Lo-Mendell-Rubin and bootstrap likelihood ratio tests were also performed. A significant *P* value indicated that the k-class model outperformed the (k−1)-class model [10]. Entropy was used as an index of classification accuracy for a given model and ranged from 0 to 1. A value of ≥0.80 was considered to signify high classification accuracy.

### Ethical Considerations

This retrospective study was conducted using fully anonymized learner data obtained from an online course platform. The study protocol was reviewed and approved by the Institutional Review Board of Sun Yat-sen University. Because of the use of deidentified retrospective data involving no more than minimal risk to participants, the study was granted exemption from full ethical review, and no specific approval number was assigned. Informed consent was waived in accordance with institutional guidelines for low-risk secondary research using anonymized data. No participants were recruited, and no compensation or incentives were provided for participation, as only anonymized, nonidentifiable secondary data were analyzed. The study adhered to the ethical principles of the Declaration of Helsinki and protected the privacy of research participants. Data were obtained with official authorization from the Wisdom Tree platform, and all learner data were anonymized and renumbered before analysis.

## Results

### Participation Rates

Academic learners maintained high participation rates across all topic tests (89.9%-91.2%), while social learners showed consistently low participation (6.7%-12.6%) and a progressive decline in engagement (Table 1).

**Table 1:** Participation rates in the different topic tests.

Topic test	Social learners (n=539), n (%)	Academic learners (n=7683), n (%)
Test 1	68 (12.6)	7005 (91.2)
Test 2	53 (9.8)	6989 (91)
Test 3	47 (8.7)	6951 (90.5)
Test 4	38 (7.1)	6937 (90.3)
Test 5	42 (7.8)	6954 (90.5)
Test 6	36 (6.7)	6910 (89.9)

### Classification

LCA identified 3 distinct participation patterns based on 6 topic tests, and the 3-class model was selected as the optimal solution (Table 2). Although Lo-Mendell-Rubin and bootstrap likelihood ratio tests supported a 4-class model, the latter generated an overly specific category with only 0.1% (9/8222) of learners that had no practical interpretive value. In large samples, these tests can detect substantively trivial divisions, leading to overextraction [13]. The emergent fourth class contributed negligible improvement in model fit (ΔAkaike information criterion <25) and, given its small size, precluded meaningful between-group comparisons, while the 3-class model had high entropy (>0.8) and meaningful, interpretable participation patterns. Therefore, consistent with recommended practices [14], the 3-class solution was prioritized on grounds of parsimony, interpretability, and statistical stability. The final classification included 3 types: participation-declining (93/8222, 1.1%), high-participation (6959/8222, 84.6%), and lowest-participation (1170/8222, 14.2%).

**Table 2:** Comparison of latent class model fit indices (N=8222).

Model	AIC^a^	BIC^b^	aBIC^c^	LMR^d^, *P* value	BLRT^e^, *P* value	Entropy	Latent class distribution, n
1-class	41,367.193	41,409.280	41,390.213	—^f^	—	1.000	8222
2-class	9925.287	10,016.477	9975.165	<.001	<.001	1.000	6974/1248^g^
3-class	9252.108	9392.399	9328.843	<.001	<.001	0.998	93/6959/1170^g^
4-class	9227.600	9416.993	9331.192	<.01	<.001	0.997	9/6958/1172/83^g^

^a^AIC: Akaike information criterion.

^b^BIC: Bayesian information criterion.

^c^aBIC: adjusted Bayesian information criterion.

^d^LMR: Lo-Mendell-Rubin.

^e^BLRT: bootstrap likelihood ratio test.

^f^Not applicable.

^g^These separated numbers represent the sample sizes of the latent classes respectively. The slash symbols are only used to separate different group values and do not indicate fractions.

**Figure 2:**
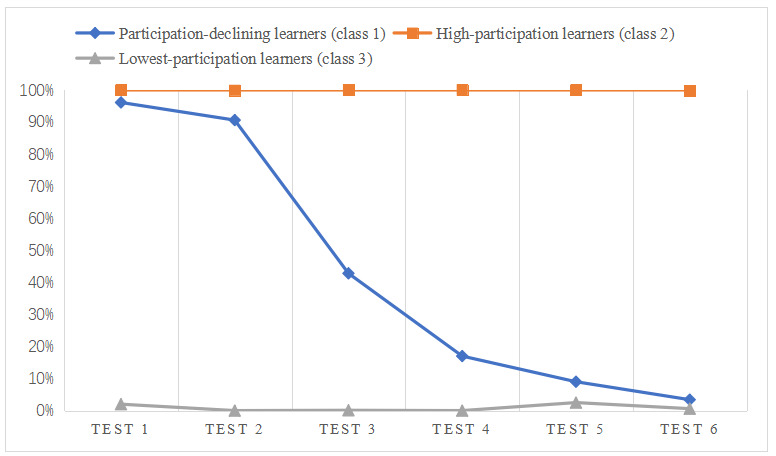
Participation probability of different types of total learners across 6 topic tests.

The participation probabilities trajectory of the 3 learner categories is shown in Figure 2. Participation-declining learners (class 1; 93/8222, 1.1%) exhibited gradually decreasing participation, with a notable decline after topic test 3. In contrast, high-participation learners (class 2; 6959/8222, 84.6%) maintained near-perfect participation rates across all tests, while lowest-participation learners (class 3; 1170/8222, 14.2%) showed minimal engagement throughout.

The distribution of learner types differed significantly between groups (*χ*^2^=2745.1; *P*<.001; Table 3). Most social learners belonged to the lowest-participation group (482/539, 89.4%), with few belonging to the high-participation (36/539, 6.7%) or participation-declining (21/539, 3.9%) groups. In contrast, most academic learners belonged to the high-participation group (6923/7683, 90.1%), with smaller proportions belonging to the lowest-participation (688/7683, 9%) and participation-declining (72/7683, 0.9%) groups.

**Table 3:** Distribution of learner types by group.

Group		Social learners (n=539), n (%)	Academic learners(n=7683), n (%)	Chi-square(*df*)	*P* value
**Learner type**				2745.1 (2)	<.001
	Participation-declining	21 (3.9)	72 (0.9)		
	High-participation	36 (6.7)	6923 (90.1)		
	Lowest-participation	482 (89.4)	688 (9)		

### Learning Performance

Academic learners significantly outperformed social learners among high-participation learners (all *P*<.05; *r*=0.06-0.10), with no significant performance differences detected between the 2 groups in the other 2 participation types (Table 4). Comparisons of subgroups with small sample sizes (n<5) were considered exploratory only, with no statistical inference value because of limited reliability and stability.

**Table 4:** Comparison of test scores between social and academic learners by learner type.

Topic test and learner type			Social learners (n=539)							Academic learners (n=7683)							Mann-Whitney *U* test				
			Mean (SD)		95% CI		n (%)		Mean (SD)			95% CI		n (%)		*Z*			*P* value		Effect size (*r*)
**Test 1**																					
	Participation-declining	89.00 (3.40)		87.21-90.79		20 (3.7)		78.84 (2.97)			78.03-79.65		69 (0.9)		−1.541			.12		−0.16	
	High-participation	74.29 (6.74)		71.98-76.60		35 (6.5)		95.56 (0.16)			95.52-95.60		6922 (90.1)		5.125			<.001		0.06	
	Lowest-participation	86.15 (4.17)		80.23-92.07		13 (2.4)		78.57 (6.45)			69.81-87.33		14 (0.2)		−0.700			.48		−0.14	
**Test 2**																					
	Participation-declining	70.00 (7.09)		66.41-73.59		18 (3.3)		75.71 (3.04)			74.94-76.48		70 (0.9)		0.721			.47		0.08	
	High-participation	69.71 (6.58)		67.49-71.93		35 (6.5)		94.63 (0.19)			94.59-94.67		6919 (90.1)		6.839			<.001		0.08	
	Lowest-participation	—^a^		0		0 (0)		—			0		0 (0)		—			—		—	
**Test 3**																					
	Participation-declining	82.42 (3.95)		79.81-85.03		11 (2)		66.00 (5.37)			64.12-67.88		30 (0.4)		−1.349			.18		−0.21	
	High-participation	71.30 (6.36)		69.18-73.42		36 (6.7)		94.40 (0.18)			94.36-94.44		6920 (90.1)		6.360			<.001		0.08	
	Lowest-participation	—		0		0 (0)		—			0		1 (0)		—			—		—	
**Test 4**																					
	Participation-declining	41.67 (41.67)		8.01-75.33		2 (0.4)		75.00 (7.41)			70.98-79.02		14 (0.2)		1.148			.25		0.29	
	High-participation	74.07 (6.63)		71.95-76.19		36 (6.7)		95.38 (0.18)			95.34-95.42		6923 (90.1)		5.192			<.001		0.06	
	Lowest-participation	—		0		0 (0)		—			—		0 (0)		—			—		—	
**Test 5**																					
	Participation-declining	66.67 (6.67)		50.11-83.23		3 (0.6)		52.00 (14.97)			38.04-65.96		5 (0.1)		−0.800			.42		−0.28	
	High-participation	58.33 (6.34)		56.29-60.37		36 (6.7)		92.27 (0.22)			92.23-92.31		6922 (90.1)		8.561			<.001		0.10	
	Lowest-participation	53.33 (6.67)		40.11-66.55		3 (0.6)		50.37 (5.05)			48.19-52.55		27 (0.4)		0.000			>.99		0.00	
**Test 6**																					
	Participation-declining	—		0		0 (0)		47.06 (8.99)			31.08-63.04		3 (0)		—			—		—	
	High-participation	57.35 (7.52)		54.51-60.19		32 (5.9)		94.57 (0.18)			94.53-94.61		6904 (89.9)		7.412			<.001		0.09	
	Lowest-participation	51.47 (17.04)		26.11-76.83		4 (0.7)		33.33 (13.73)			12.11-54.55		3 (0)		−0.535			.59		−0.20	

^a^Insufficient data for analysis (n<5).

## Discussion

### Principal Findings

As an observational study, this research explored factors associated with performance disparities between social and academic learners in a rehabilitation nursing MOOC from the perspective of participation type distribution; causal relationships between the investigated variables cannot be established, and only associative correlations were identified. There were three key findings: (1) divergent participation type distributions between social and academic learners; (2) superior performance of academic learners in the high-participation subgroup; and (3) no significant performance differences in non–high-participation subgroups.

Most notably, the 2 groups showed starkly divergent participation type distributions—the core factor associated with their overall performance gap: more than 90% of academic learners belonged to the high-participation group, whereas nearly 90% of social learners belonged to the lowest-participation group. This underscores the pivotal impact of learner background and learning environment on participation patterns [15]. As nursing undergraduates, academic learners sustained high participation through teacher supervision, peer learning, and credit pressure—a robust external support system for continuous engagement, consistent with Uijl et al [16], who found interpersonal connections and structured learning arrangements to enhance learner interaction and participation. In contrast, social learners lacked such external constraints and institutional support despite greater autonomy, leaving them prone to low participation or midcourse dropout [17]. These patterns align with prior LCA of nursing MOOCs [9]. Wang et al [9] also identified committed learners as the highest-performing group, with no significant performance differences across noncommitted types. While Wang et al [9] distinguished 4 classes among course completers, this analysis of all registrants yielded 3, a discrepancy likely attributable to sample composition differences [18]. This work extends prior research by demonstrating that academic learners’ performance advantage stems from their overrepresentation in the committed learner group.

In addition, academic learners’ high-participation subgroup outperformed social counterparts across all 6 topic tests (all *P*<.001) with trivial to small effect sizes (*r*=0.06-0.10) [19]. This large-sample research characteristic arose because the sizable academic high-participation cohort (n≥6900) heightened the Mann-Whitney *U* test sensitivity, detecting minor mean differences as significant [20]. In contrast, participation-declining and lowest-participation subgroups (mostly n<50) showed no statistical score differences and similarly trivial to small effect sizes, with their disparities bearing neither statistical nor practical meaning. This confirms the high-participation group as the core carrier of performance differences between cohorts, validating our design centered on participation type distribution.

The far higher proportion of high-participation learners among academic groups, and their stable, sustained engagement, drives the superior overall performance of academic learners: such learners exhibit strong engagement and self-regulation, enabling systematic task completion, active course participation, and in-depth knowledge acquisition and transfer. Conversely, the low proportion of high-participation learners among social learners underpins their poorer performance. While prior research identified high-participation learners as the highest-performing subgroup [8,9], our findings further reveal that background-based disparities in their proportion decisively shape learning outcomes, highlighting participation composition as central to explaining performance differences. Consistent statistical significance in the high-participation group’s gap confirms academic learners’ stable advantage, a strength derived from institutional external support rather than inherent learning ability gaps [21].

Our analysis also showed that both academic and social learner groups featured a certain proportion of participation-declining and lowest-participation learners, although there were no significant differences between these learners in academic performance. This finding is consistent with the early classifications of MOOC learner types. For example, Reich [22] and Gilligan et al [23] identified low-participation groups, including bystander learners and early dropout learners. Subsequent studies have confirmed that these groups are widespread and achieve poor learning outcomes [22-24]. This may be due to the general lack of internal learning motivation and continuous engagement among such learners. This aligns with self-determination theory, which posits that when internal motivation is insufficient, the effect of external incentives is also greatly reduced, making it difficult to drive effective learning behaviors [5]. These data also indicate that once learners lack continuous engagement, it is difficult for their academic performance to reach the desired level regardless of their background. Therefore, future development and application of MOOCs should involve incentive mechanisms, emotional support, and learning analytics to increase student participation and reduce the proportion of participation-declining and lowest-participation learners.

The predominance of committed learners among academic students (6923/7683, 90.1% vs 36/539, 6.7% in social learners) suggests that participation type may serve as an intervening variable in the pathway between learner background and performance—a finding consistent with recent MOOC research demonstrating that engagement patterns link learner characteristics to outcomes [25]. However, this observational finding is hypothesis-generating only. Academic learners’ superior performance may equally reflect offline institutional support absent for social learners, and unmeasured mediator-outcome confounding cannot be ruled out [26]. Future causal mediation studies with comprehensive covariate measurement are needed to test whether participation patterns represent a genuine pathway.

### Limitations

This study has several limitations. First, the lack of demographic data (eg, age and prior educational background) from the online platform limited our ability to control for potential confounding variables in the analysis. Second, some subgroups had extremely small sample sizes (n<5), which reduced the statistical power of relevant comparisons and limited their generalizability. These results should therefore be considered exploratory and require further validation in future studies with larger samples.

### Conclusions

This study identified 3 distinct participation types (participation-declining, high-participation, and lowest-participation) among rehabilitation nursing MOOC learners. Academic learners predominantly belonged to the high-participation group, while social learners predominantly belonged to the lowest-participation group. The performance advantage of academic learners was primarily associated with the larger proportion of high-participation learners in their group, and academic learners significantly outperformed social learners within this subgroup. No significant performance differences were found between the 2 groups in the participation-declining and lowest-participation types, confirming that sustained participation is key to improving MOOC learning outcomes. These findings provide empirical evidence for nursing MOOC teaching design, suggesting that instructors should prioritize sustaining learner participation and implement tailored support strategies for voluntary social learners to enhance their engagement and persistence, thereby narrowing the performance gap between the 2 groups.

## Data Availability

The data that support the findings of this study are available from the Wisdom Tree online education platform [11], although restrictions apply to the availability of these data, which were used under license for this study. Aggregated data (eg, participation rates by learner group, mean test scores by participation class, and latent class analysis model fit indices) are available from the corresponding author upon reasonable request and with permission from the Wisdom Tree online education platform. Raw learner log data cannot be shared due to platform data privacy policies.

## Abbreviations

LCA: latent class analysis
MOOC: massive open online course
